# Use of Self-Efficacy Scale in Mass Casualty Incidents During Drill Exercises

**DOI:** 10.1186/s12913-024-11175-w

**Published:** 2024-06-18

**Authors:** María Carmen Cardós-Alonso, Miguel Inzunza, Lina Gyllencreutz, Salvador Espinosa, Tatiana Vázquez, Maria Aranzazu Fernandez, Alberto Blanco, Ana María Cintora-Sanz

**Affiliations:** 1 Emergency Medical Service of the Community of Madrid (SUMMA112), Madrid, Spain; 2 Faculty of Nursing, Physiotherapy and Podiatry, Complutense University of Madrid, Madrid, Spain; 3 Unit of Police Work /Research Unit, Umeå University, Umeå, Sweden; 4 Department of Nursing, Umeå University, Umeå, Sweden; 5 Department of Diagnostics and Intervention, Umeå University, Umeå, Sweden

**Keywords:** Disaster training, Mass casualty incidents, Emergency medical services, Simulation, Self-efficacy

## Abstract

**Introduction:**

Medical First Responders (MFRs) in the emergency department SUMMA 112 are tasked with handling the initial management of Mass Casualty Incidents (MCI) and building response capabilities. Training plays a crucial role in preparing these responders for effective disaster management. Yet, evaluating the impact of such training poses challenges since true competency can only be proven amid a major event. As a substitute gauge for training effectiveness, self-efficacy has been suggested.

**Objective:**

The purpose of this study is to employ a pre- and post-test assessment of changes in perceived self-efficacy among MFRs following an intervention focused on the initial management of MCI. It also aimed to evaluate a self-efficacy instrument for its validity and reliability in this type of training.

**Method:**

In this study, we used a pretest (time 1 = T1) – post-test (time 2 = T2) design to evaluate how self-efficacy changed after a training intervention with 201 MFRs in initial MCI management. ANOVA within-subjects and between subjects analyses were used.

**Results:**

The findings reveal a noteworthy change in self-efficacy before and after training among the 201 participants. This suggests that the training intervention positively affected participants’ perceived capabilities to handle complex situations like MCI.

**Conclusion:**

The results allow us to recommend a training program with theory components together with practical workshops and live, large-scale simulation exercises for the training of medical first responders in MCI, as it significantly increases their perception of the level of self-efficacy for developing competencies associated with disaster response.

**Supplementary Information:**

The online version contains supplementary material available at 10.1186/s12913-024-11175-w.

## Introduction

Mass Casualty Incidents (MCI) are defined by the local health system’s ability to address the initial health needs of victims, influenced by the ratio between victims and available resources. These incidents, involving multiple patients, can temporarily overwhelm and collapse the Emergency Medical Response (EMR) [1, 2]. MCI’s inherent complexity, influenced by factors like incident type, casualty numbers, resources availability, timing and weather conditions, and triage system used [3], makes developing a curriculum challenging for teaching Medical First Responders (MFRs) how to handle victims in this environment [4].

The chaos of MCIs creates stressful situations for professionals, which can lead to loss of situational awareness, fixation errors, and hindered communication, all affecting decision-making and patient outcomes. Experts often struggle to explain their decision-making process during MCI care [5]. Studies suggest that stress levels among emergency professionals vary and impact them differently [6]. Additionally, training on-site can improve their subsequent performance [7, 8].

Training in safe scenarios improves care outcomes, reduces coping stress, results in better decision-making and decreases emotional impact, all of which increases the quality of care and patient safety [9], as well as changing the perception of their work [10].

Simulation for training healthcare workers in emergencies is based on the ability to reproduce rare events in a safe environment for patients and professionals ([11, 12], Kim et al. 2020). In addition, it is also a significant advantage in the possibility of working on technical and non-technical or relational skills, with results being superior to passive learning such as reading or lectures [13]. Simulation-based training is increasingly used in emergency and disaster management to acquire the necessary knowledge, skills and experience [14].

Kolb’s Experiential Learning Theory for adult learning argues that acquired skills, if not practised, decrease in effectiveness after 24 months [15]. Thus, it is important that the skills acquired and trained are becoming automated over time and embedded in the individual, as one of the key elements is the subsequent analysis, generating new concepts that can be put into practice in the next situation [5, 12]. This characteristic makes it difficult for practitioners to describe the specific behaviours that make crisis resolution successful [16]. More specific related to acute situations, decision-making in emergencies is very difficult to study as it is an internal process that occurs rapidly and carries a great deal of responsibility [17].

Until recently, MCI training was carried out through table-top exercises and large-scale drills, demanding considerable human resources and equipment [18]. However, frequent repetition of such training is both unsafe and expensive. Due to the infrequent provision of training in disaster response, it is not surprising that many healthcare professionals, including MFRs, perceive their preparedness as inadequate [19, 20]. Research indicates that higher frequency and quality of training directly correlate with better disaster preparedness [21].

Two models of interactive (learning-by-doing) training in emergency preparedness have been developed in Sweden: practical field exercises or “tabletop” exercises. In this study [22], they used the standardized MAss Casualty SIMulation (MACSIM®) training model, a scientifically validated simulation system of “tabletop exercises” for the training of hospital, prehospital and collaborating agency medical personnel. The study demonstrates that this type of simulation exercise is useful for training healthcare personnel, as well as helping to develop emergency plans or revealing deficiencies in existing ones, although field exercises should also be conducted. Both models have certain advantages or disadvantages. A challenge for emergency planners is to choose the most appropriate model to achieve the best learning outcomes for participants. Drills should be conducted to ensure the feasibility of the evacuation plan.

Several models for evaluating training programs are available [23]. The Kirkpatrick model is widely used and consists of four levels: reaction, learning, behavior, and results (Annex I). Each level builds upon the previous one, with higher levels of evaluation recommended only after success is demonstrated at lower levels. However, this progressive evaluation process can become increasingly complex and resource-intensive [24, 25].

Another model to evaluate preparedness is the self-concept that is a concept that is frequently used in research [25] and “refers to a person’s self-perceptions concerning important aspects of life” [26]. The process of transitioning from self-concept to self-efficacy involves identifying and recognizing particular skills and abilities within one’s overall self-perception. Subsequently, it focuses on cultivating a belief in the ability to employ those skills effectively to achieve specific goals. A positive self-efficacy is the key for emergency professionals to manage the different phases of multi-victim incident resolution. Measuring participants’ self-efficacy is one approach to evaluating the impact of a training intervention. Self-efficacy indirectly measures the training’s effect on improving healthcare skills, providing insights into the potential impact of an educational intervention on subsequent clinical practice [27, 28].

Each individual’s behavioural choices are based on his or her self-efficacy expectations. Training in technical skills is necessary, but not sufficient, to perform well. Technique is a means to achieve certain outcomes, but in itself does not constitute an outcome expectation. In fact, “effectiveness in behaviour requires continuous improvisation of skills to master the continually changing circumstances of the environment, most of which are made up of ambiguous, unpredictable and often stressful elements” ([29], p. 416 [30]). Self-efficacy is a dynamic and context-specific construct considering individuals’ perceptions of their capabilities in a particular situation.

The self-efficacy belief mediates the impact of environmental conditions on the person’s behavior; that is, those who possess a high level of self-efficacy expectations can cope more successfully with these conditions, while generating behavior that in one way or another can also modify these conditions. In MCIs, individuals with high self-efficacy are more likely to engage in problem-solving behaviours, persist in adversity, and collaborate effectively. In contrast, those with low self-efficacy may experience heightened anxiety, struggle with decision-making, and exhibit decreased resilience [31]. According to these statements students with a higher degree of self-efficacy following Kolb’s experiential model would have greater growth in each of the turns of the circle, with its four phases: experiencing, reflecting, theorizing and acting [32]. Self-efficacy does not tell us how many of these lives have been saved thanks to the simulation, but it speaks to us about a change in the learner’s behavior, which would correspond to level 3 of evaluation, within Kirkpatrick’s [33, 34] evaluative model (Annex I).

Research of this kind is fundamental because academic self-efficacy has been shown to predict cognitive engagement and academic achievement in various educational contexts [30].

## Objectives

This study aims to employ a pre-and post-test to assess the alterations in self-efficacy among MFRs after a training intervention focused on the initial management of an MCI. The purpose was also to determine a self-efficacy instrument regarding validity and reliability.

## Methods

### Design

This study used a pretest (time 1 = T1) – post-test (time 2 = T2) design to evaluate how self-efficacy changed after a training intervention with MFRs in initial MCI management. This scale was previously analyzed for validity and reliability through a Confirmatory Factor Analysis (CFA) with the maximum likelihood method. Subsequently, Cronbach’s alpha was calculated to obtain the reliability of the factors and the entire scale.

### Training intervention

During the year 2023, between February and October, eight iterations of an MCI training program were conducted. This program is designed for employees working at the Prehospital Emergency Medical Service of Madrid Community (SUMMA 112) and stands as an integral component within the training framework for MFRs.

061 was created by the Special Emergency Service of Madrid (SEU) in January 1964. Its birth 40 years ago constituted one of the pioneering experiences in Europe in the implementation of out-of-hospital emergency medical care services. It has undergone changes and mergers until today’s SUMMA 112.

SUMMA 112 is the Emergency Medical Service of the Community of Madrid, handling out-of-hospital emergencies with a vast network of resources. Its functions include:

- Managing health-related phone calls.
- Providing healthcare in MCI.
- Coordinating critical patient transfers.

SUMMA 112 is not just a reactive service. It takes a proactive approach, engaging in epidemiological alerts, international health missions, and organ transplant activities. The organization conducts external training and research in emergency and disaster management, always staying ahead of the curve. Staffed by emergency physicians, nurses, and technicians focused on first responder needs, SUMMA 112 supports end-user testing, identifies training gaps, and evaluates technologies with health experts in various scenarios. This proactive stance ensures that SUMMA 112 is always prepared to handle any emergency situation.

The program comprises both theoretical instruction and practical features.

The theoretical part focuses on theoretical content delivered through online modalities, which participants must complete and pass before the practical component. The practical component includes practical workshops and culminates in live drill exercises (Annex I).

MCI trainers with the necessary training and accreditation deliver this theoretical and practical component.

The course is divided into two days. The first day, as can be seen in Annex II, consists of a five-hour theoretical session divided into several lessons: review of the procedure for multiple victim incidents of the Madrid emergency service (definition of MCI and the criteria for its activation), roles of the different responders, colors of the vests they must wear (to be differentiated during the action), communications between interveners (communication channels to be used, radio language, communications procedure), triage (types of triage, triage backpack and its contents, triage cards, life-saving measures using tourniquets, oropharyngeal cannulas, hemostats, etc.

At the end of the day, doubts will be answered and finally a theoretical exam will be performed. The second day consists of carrying out 3 simultaneous workshops, which the students must go through. After the workshops, two MCI exercises will take place, where students will be able to practice what they have learned. At the beginning of the day, the researchers explain the self-efficacy study to the trainees and collect the consent inform. In the first workshop, the procedure for an MCI action in the SUMMA 112 service is reviewed. It consists of a dynamic using a whiteboard and magnetic markers that represent the different participants in a MCI. The zoning of the incident will be carried out in three areas: intervention zone (firefighters and rescue teams), relief zone (triage zone, advanced health post, stretcher wheel) and base zone (advanced command post, waiting area of resources).

In the second workshop, the different roles that the MFRs will take when arriving at the MCI are explained, as well as the different vests they will have to put on to be differentiated during the action. The roles in the MCI will be: head commander (in charge of all the medical teams) (red vest), triage commander (blue vest), commander in charge of resources (orange vest),commander of the advanced medical post (green vest), responsible for affiliation (green vest), responsible for evacuation and charge (green vest), responsible of the communications of the advance medical post (black vest), and commander in charge of light or green casualties (green vest). In addition to this, in this workshop the triage card and the triage backpack are shown (how to fill the triage card, life saving maneuvers, contents of the backpack).. Our service uses the START method for the first triage and the revised trauma score for the second triage.

The last workshop emphasises using communication, using the correct channel, and providing clear and concise information. After the workshops, the drill exercise is based on two scenarios, each lasting approximately 45 min. The first is a bus accident with 15 victims, and the second is a building explosion with 20 victims. In this exercises, the trainees simulate everything that would happen from the beginning in a MCI, putting on the safety equipment, using the communications devices, triaging patients, transferring them to the advanced medical post, prioritizing their evacuation and so on…Before each exercise, there will be a debriefing. At the end of the second day, the researchers will give the questionaries of the self-efficacy study to the trainees.

### Participants

Employees enrolled in the MCI training program were selected for participation. The sampling approach was convenience sampling from the SUMMA 112 Emergency Medical Service. The study successfully recruited a comprehensive cohort comprising 201 participants. The age variable was transformed to have three categories (40 or below, 41 to 55, 56, and above), gender (female, male), occupation (doctor, nurse, and technician), and experience transformed to have three categories (10 years or less, 11 to 20 years, and 21 years or more). The sample description is composed of 42.8% women and 57.2% men. Among them we find 18.4% of physicians, 26.9% of nurses and 54.7% of technicians. In relation to age, 25.4% were 40 years of age or younger, 46.3% were between 41–50 years of age, and 28.4% were 51 years of age or older. Finally, 40.5% had 10 years of work experience in emergencies or less, 38% had between 11 and 20 years and 21.5% had more than 20 years.

### Instrument

The instrument utilized in this study is the “Self-efficacy scale for first responders in MCI” (SESMCI), developed and validated by Cardós et al. [35], depicted in Table 1. The SESMCI is produced from the existing scales “The Disaster Preparedness Perception Scale in Nurses” (DPPSN) [33] and “The Disaster Response Self-Efficacy Scale” (DRSES) Li et al. [36]. These scales have been validated by Kim [6], Cruz et al. [37], and Toraman et al. [38] in various countries and with different MCI training methods [39]. Additionally, the SESMCI instrument has been influenced by the “Learner Evaluation Questionnaire (LEQ)”, originally designed to assess medical students’ attitudes toward the curriculum [40]. The SESMCI instrument employs a 6-point Likert scale to measure self-efficacy before and after the simulation exercise, with the scale ranging from 1 (No Trust) to 6 (Total Confidence).

**Table 1:** Self-efficacy scale for first responders in mass casualty incidents (SESMCI)

**My degree of confidence in performing the following actions is…**		
**Ítems**		**Degree of confidence**
1.	Mental reminder to fulfil the MCI intervention protocol as I approach the scene.	1 - 2 - 3 - 4 - 5 - 6
2.	Proper use of self-protective techniques and/or material? at the scene (in order to prevent intrinsic and extrinsic hazards).	1 - 2 - 3 - 4 - 5 - 6
3.	Coordination with the team members to initiate appropriate actions, according to my role in the MCI. (Distribution of tasks).	1 - 2 - 3 - 4 - 5 - 6
4.	Use communication devices correctly according to MCI procedures in each country.	1 - 2 - 3 - 4 - 5 - 6
5.	Manage the deployment of resources effective and efficiently.	1 - 2 - 3 - 4 - 5 - 6
6.	Installation and supervision of working areas (zoning) and deployment of eventual structures.	1 - 2 - 3 - 4 - 5 - 6
7.	Perform the first triage (classification) within the allocated time and MCI procedures.	1 - 2 - 3 - 4 - 5 - 6
8.	Perform life-saving manoeuvres when needed (according to MCI procedures).	1 - 2 - 3 - 4 - 5 - 6
9.	Refer each patient to the assigned location according to priority.	1 - 2 - 3 - 4 - 5 - 6
10.	Apply the treatment prescribed at the medical post.	1 - 2 - 3 - 4 - 5 - 6
11.	Perform the evacuation or second triage correctly according to the MCI protocol. (referral to utility centre).	1 - 2 - 3 - 4 - 5 - 6
12.	Evacuate the victims correctly according to the MCI protocol.	1 - 2 - 3 - 4 - 5 - 6
13.	Perform a self-assessment with my peers in a debriefing afterwards.	1 - 2 - 3 - 4 - 5 - 6

### Data collection

Data was collected on two occasions, referred to as time 1 = T1 and time 2 = T2, and the same data collection method was employed on both occasions. The technique involved providing each participant, after informing them of the importance of their participation and the possibility of withdrawing from the study whenever they wish to do so, with a questionnaire to fill in before the practical workshops and drill exercises on day 2. The questionnaire included background questions and the 13 questions comprising the self-efficacy instrument. After completing the questionnaire (T1), the participant participated in the practical workshops and drill exercises. Immediately following the training, the participant completed the same questionnaire (T2). The questionnaires were coded with numbers to ensure they could be linked.

### Data analysis

The tools used for analysis at item level, descriptive analysis and group differences was SPSS [41] version 28.0.1. The analysis concerning the latent variable models investigating construct validity according to fit indices were performed with Mplus version 8 (Muthén and Muthén, 1998–2017).

A global descriptive analysis was conducted at the item level between T1 and T2 using the Wilcoxon Signed Ranks Test. It was found that in all items, the mean score of the Likert scale increased by approximately one point and reported a *p*-value < 0.001, which confirms the very significant increase in the perception of self-efficacy after the practical workshop and brief exercises of MCI (Table 2).

**Table 2:** Global statistical analysis (Wilcoxon Signed Ranks Test)

	Stockings Inc	Climbing	Fall	Equal	Wilcoxon *p*
POST1 - PRE1	0,990	150	7	44	< 0.001
POST2 - PRE2	0,811	123	7	71	
POST3 - PRE3	0,905	132	13	56	
POST4 - PRE4	1,060	145	12	44	
POST5 - PRE5	1,060	147	10	44	
POST6 - PRE6	1,129	148	7	46	
POST7 - PRE7	1,080	136	7	58	
POST8 - PRE8	0,652	107	14	80	
POST9 - PRE9	0,831	131	10	60	
POST10 - PRE10	0,736	117	12	72	
POST11 - PRE11	0,925	134	14	53	
POST12 - PRE12	1,035	143	9	49	
POST13 - PRE13	0,876	122	7	72	

The study encompasses the assessment of sociodemographic data, including age, gender, profession, and years of experience within the emergency services.

The validity and reliability of the dependent variable self-efficacy were investigated with latent variable modelling by conducting a confirmative factor analysis (CFA). The fit of the developed model was assessed according to several suggested fit indices with recommended thresholds. An insignificant chi square *p* > 0.05; CFI with a minimum of 0.90 indicates an acceptable model; RMSEA of 0.08 or lower indicates a model with a satisfactory fit; SRMR of 0.08 or lower for a model with an adequate fit [42, 43, 44]. The omega coefficient was used to assess reliability following the recommendations for the reliability of latent variable models [45]. A repeated measures ANOVA within-subjects and between subjects was performed to investigate the effect of the intervention [46, 47] and when studying group differences based on age, gender, occupation and experience with a significance level *p* set at < 0.05.

Mann-Whitney U-test for independent samples was applied to investigate if there were gender differences in their perception of self-efficacy.

Independent-Samples Kruskal-Wallis Test was performed to investigate the differences between doctors, nurses, and technicians concerning their self-efficacy.

## Results

### Dependent variable

**Fig. 1:**
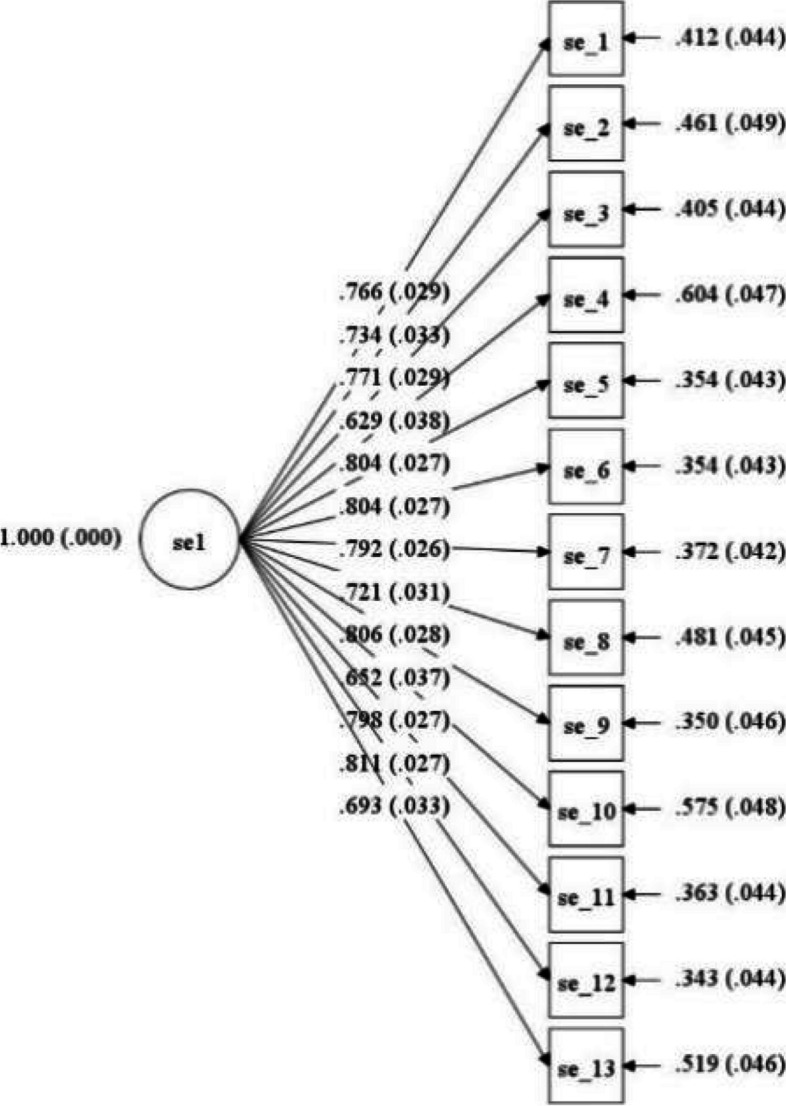
Standardized estimates for a 1-dimensional 13-item model at T1. Pre – post measurement

The 13-item measure of Self-efficacy (Fig. 1) was useful with the current sample of participants.

Within this cohort, individuals aged up to 40 years constituted 25.4%, those in the 41–50 age bracket accounted for 46.3%, showcasing the highest mean age frequency, and individuals aged > 50 years comprised 28.4%. Regarding gender distribution, the cohort encompassed 86 females and 115 males. Additionally, participants were stratified by job category, indicating that technicians constituted the majority at 54.7%, followed by nurses at 26.9%, and doctors at 18.4% (Table 3). The calculated mean work experience across all participants was 14.35 years.

**Table 3:**
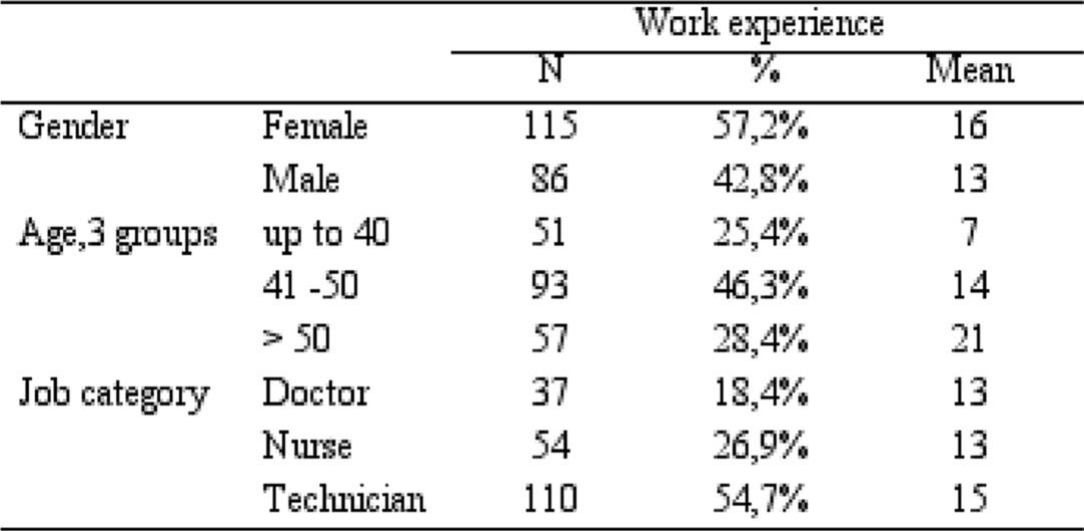
Demographics of participants

CFA analyses of a 1-dimensional model showed a weak fit to the data at time 1 according to some of the selected fit indices, significant chi-square (65) = 252.46, *p* < 0.01; CFI = 0.87; RMSEA = 0.12; SRMR = 0.06. At time 2 most fit indices showed an acceptable fit to the data, significant chi-square (65) = 155.17, *p* < 0.01; CFI = 0.94; RMSEA = 0.08; SRMR = 0.04. Loadings were all significant and above 0.60 at both measurements. Reliability calculated as Omega was 0.94 at time 1 and 0.95 at time 2. Composite scores for time 1 and time 2 were developed from the 13 items and ranged at time 1 from 18–78 and at time 2 from 22–78, where a higher score indicated a higher level of Self-efficacy.

Our findings indicate a significant difference in Self-Efficacy pre-post training with a sample of n = 201 participants. A one-way within-subjects ANOVA test showed F (1,200) = 369.893, *p* < 0.01, η2 = 0.65. The following observed means were noted at the different time points: T1 (M = 52.40, SD = 11.15) and T2 (M = 64.49, SD = 9.70).

**Fig. 2:**
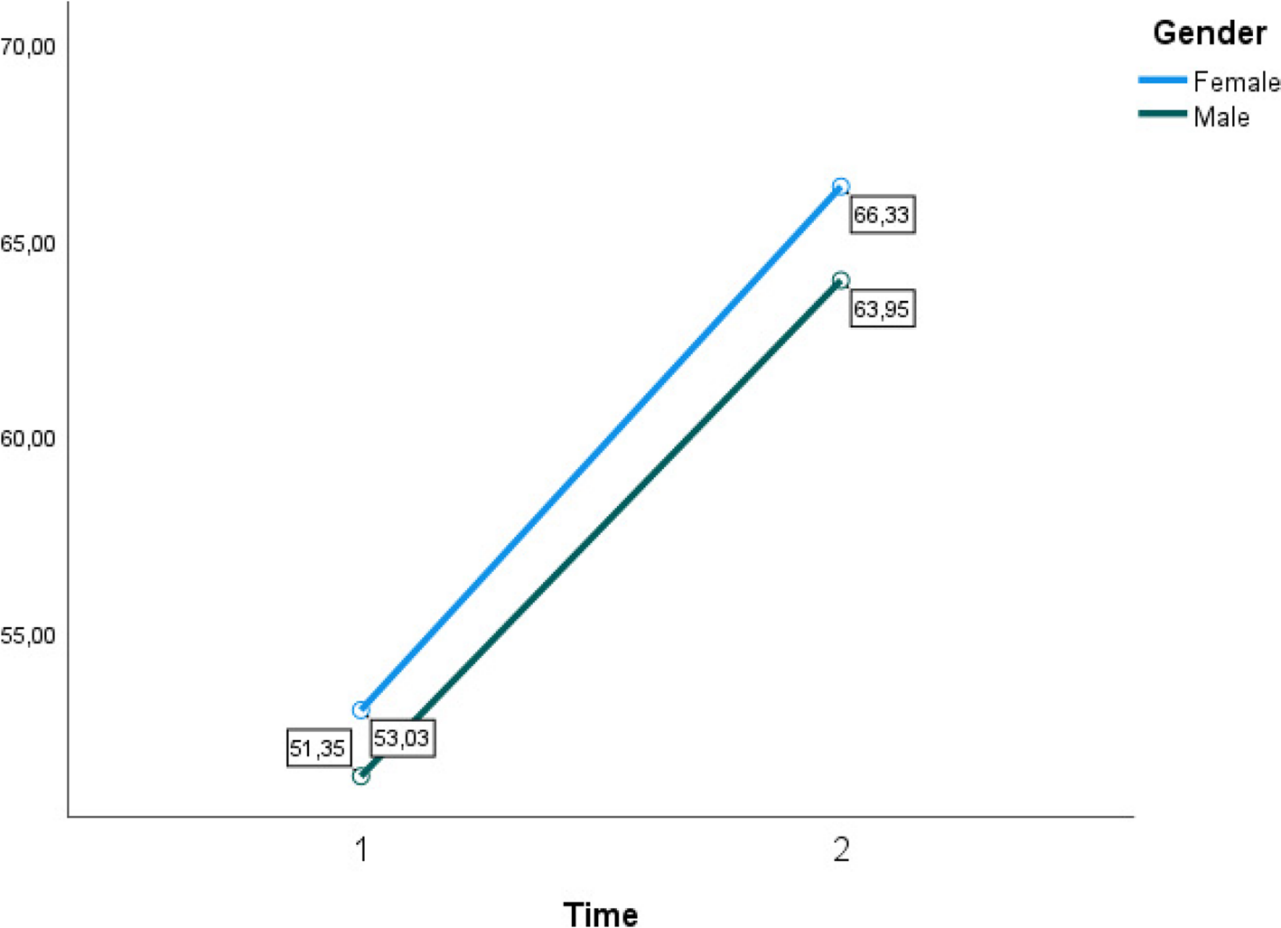
Estimated marginal means for group variable gender time 1 and time 2

**Fig. 3:**
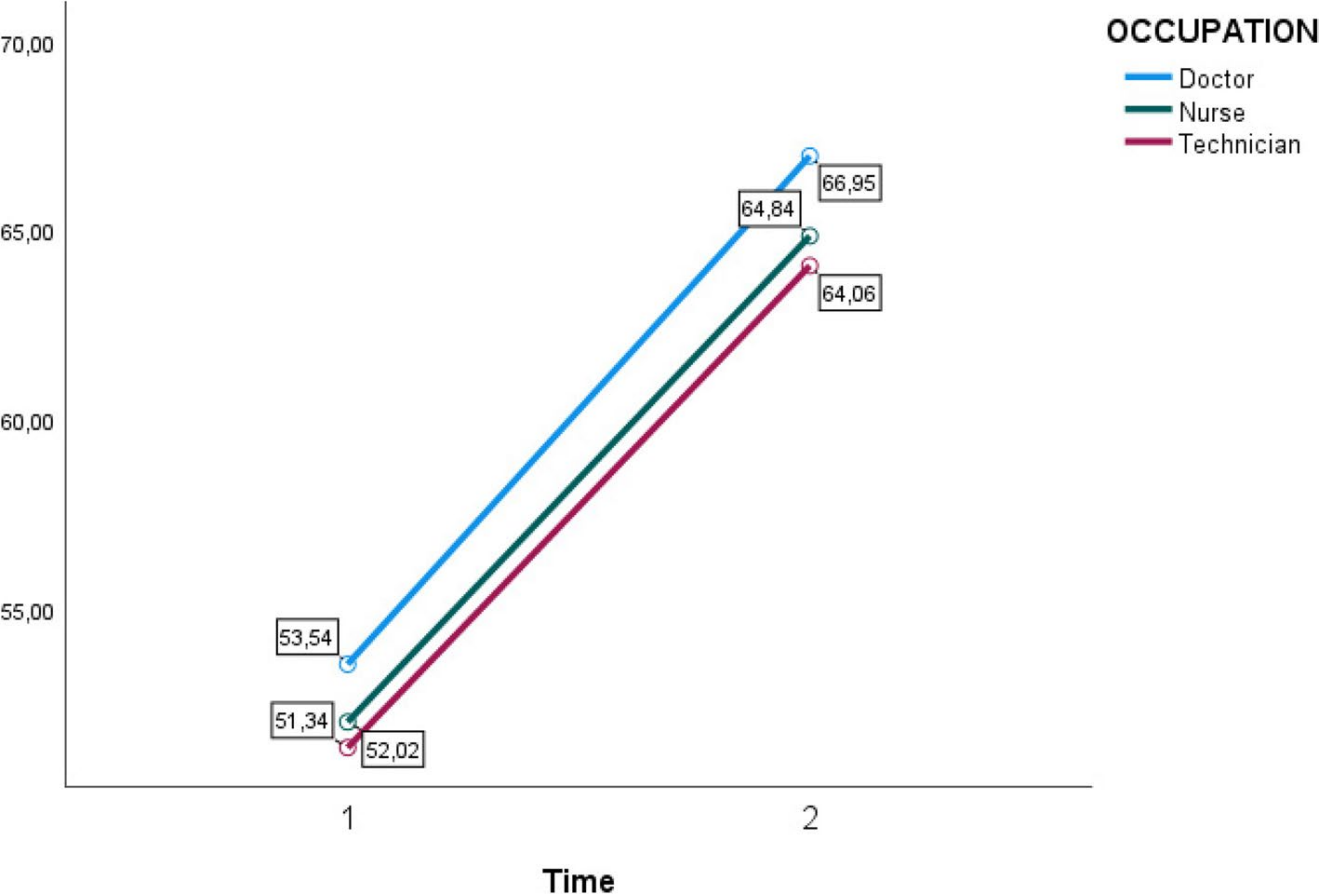
Estimated marginal means for group variable occupation time 1 and time 2

**Fig. 4:**
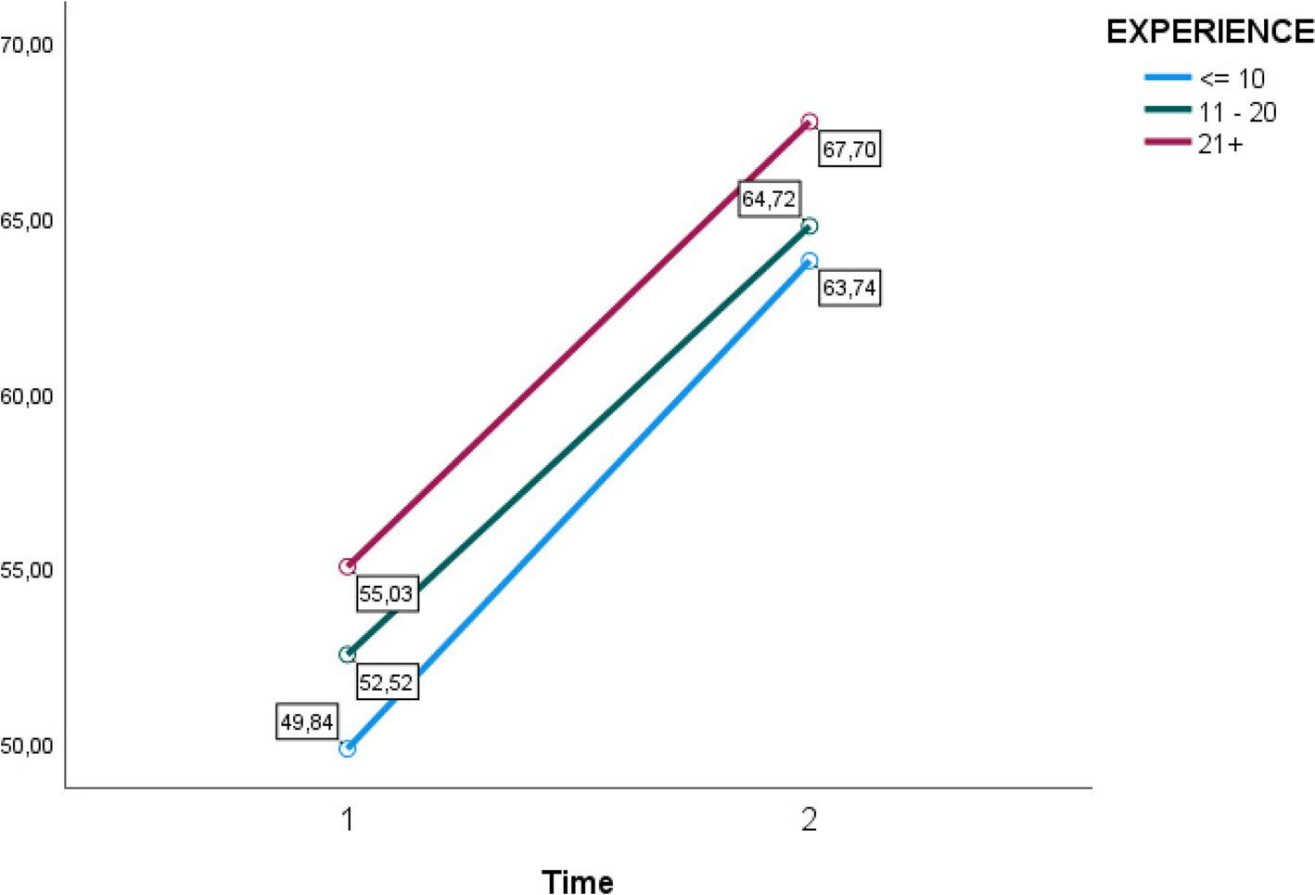
Estimated marginal means for group variable experience time 1 and time 2

**Fig. 5:**
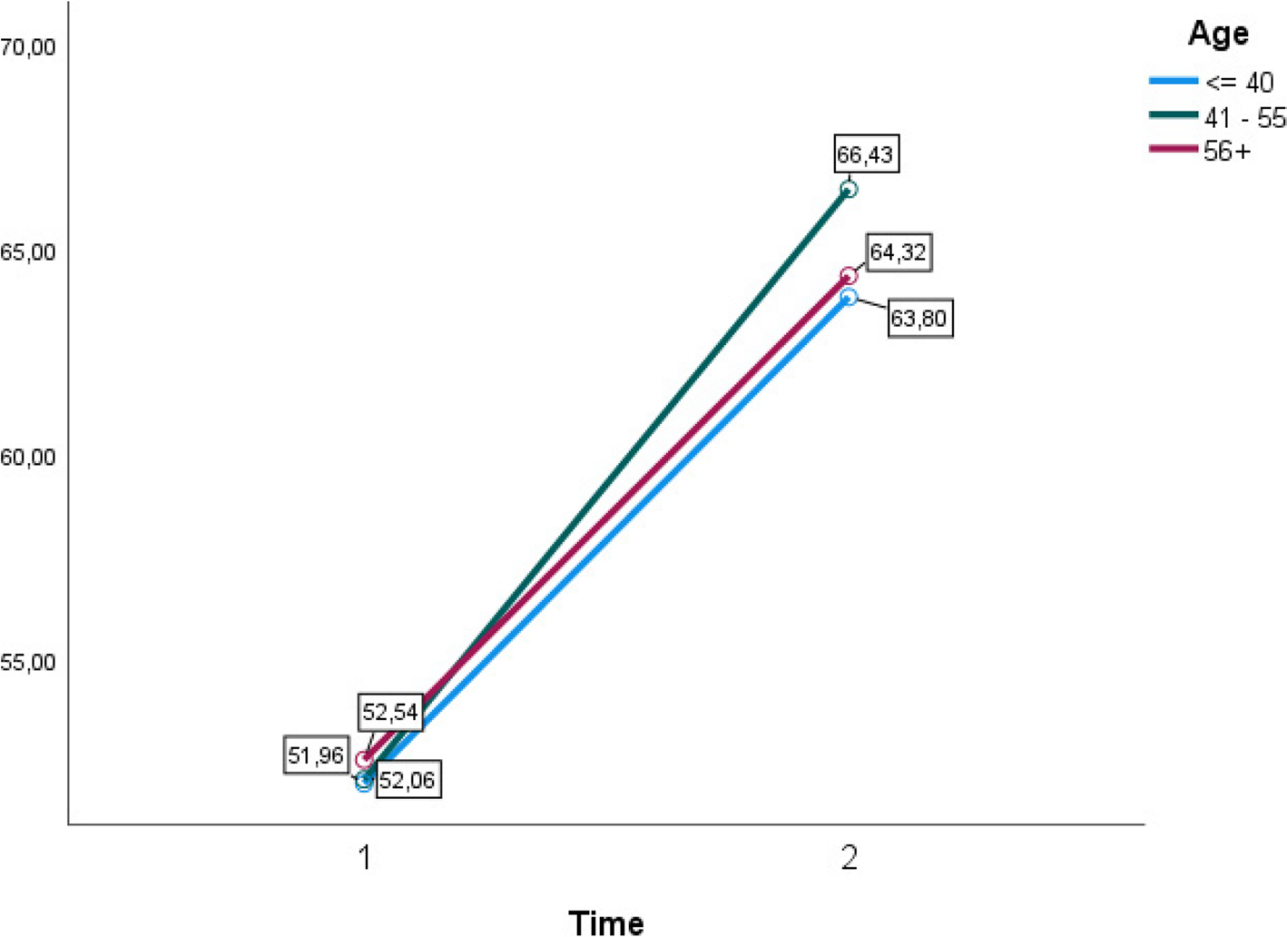
Estimated marginal means for group variable age time 1 and time 2

When incorporating group variables, no significant interaction effect could be noted based on any of these variables (Figs. 2, 3, 4 and 5). A within and between-subjects design ANOVA test showed *F* (1,62) = 123.33, *p* = 0.01, η2 = 0.43. The following observed marginal means were noted with the included variables (Figs. 2, 3, 4 and 5).

To conclude, there is a significant difference between time 1 and time 2 with a considerable effect, η2 = 0.65, η2 = 0.43. There were no significant interaction effects when including group variables in the model. Assumptions of equality of covariance matrices (homogeneity of intercorrelations) have been controlled with an insignificant Box’s test.

The findings show no significant differences between genders in improving perceived self-efficacy. However, when administering the prescribed treatment at the medical post, a p-value of 0.008 was obtained, indicating a more substantial increase in self-efficacy among men than women.

Concerning results about the differences between doctors, nurses, and technicians, our findings reveal statistically significant differences in 4 of the 13 items. Specifically, the self-efficacy of doctors and nurses significantly increases in performing/supervising the deployment of means and resources effectively and efficiently. In contrast, technicians perceive themselves as less self-effective in this task. Furthermore, variations were observed in applying the prescribed treatment at the advanced medical posts. Significant differences were observed among technicians who considered themselves more prepared after the simulations than the other professional categories. According to the MCI protocol, evacuating victims revealed significant differences between doctors and technicians. However, no notable distinctions were observed between nurses and the other two professional categories in this aspect.

## Discussion

Regarding the teaching methodology used, following the simulation zones [48], for the evaluation in behavioral change or in the perception of behavioral change that would be a level 3 of Kirkpatrick [34], simulation scenarios designed for zones 2 and 3 are used, which allow giving answers to how and why. But for this it is essential that the student has been able to acquire and practice the knowledge, and it is for this reason that the course had a theoretical part of knowledge acquisition, a practical part where the different skills are trained, individually (zone 1) or combining the different skills (zone 2) and a part of simulations where the human factors are also trained (zone 3) (Annex I).

In the dynamic realm of medical education, the concept of self-efficacy has garnered significant attention as a driving force behind learner motivation, academic success, and professional development. Stemming from Bandura’s social cognitive theory, self-efficacy reflects individuals’ beliefs in their capabilities to perform specific tasks [49, 50, 51, 52].

The results indicate that the 13-item self-efficacy scale proved to be a valuable tool for assessing self-efficacy among participants in this study, where we used training methods using didactic tools simulating victims and scenarios without new technologies. This aligns with previous research in medical education that has explored various instruments to enhance self-efficacy (cf. [53, 54, 55, 56, 57]).

The result from this study, demonstrate that a comprehensive MCI training program, which includes theoretical instruction, practical workshops, and a simulation-based training exercise, significantly increased overall self-efficacy and management skills in the context of patient treatment, coordination, and communication skills during simulated MCI. This finding aligns with previous research that underscores the value of simulation-based training promising to influence self-efficacy beliefs positively (cf. [58, 59]). Additionally, these results suggests that such a training method have the potential to boost the self-confidence of healthcare emergency professionals, bolstering their ability to perform tasks effectively, maintain resilience in challenging scenarios, and persevere through obstacles. This aligns with more than 30 years of self-efficacy research indicating that merely possessing knowledge and skills is insufficient for ensuring that students will apply them when needed. Instead, both “the skill and the will” are necessary for medical students to function successfully in dynamic clinical contexts [52, 53, 60]. Therefore, educators are encouraged to adopt instructional approaches that fosters competence and promote the growth of self-efficacy. Consequently, our findings contribute an effective training program to elevate self-efficacy, thereby enriching evidence-based educational strategies. Ultimately, this empowers trainees to navigate the complex and demanding landscape of disaster response medical training.

The findings reveal a noteworthy change in self-efficacy before and after training among the 201 participants, as evident by Cohen effect sizes > 0.14 which suggest a large effect [61]. However, the absence of significant interaction effects for gender, occupation, experience, or age indicates that these factors did not significantly influence the observed outcomes. This suggests that the training intervention positively affected all participants’ perceived capabilities to handle complex situations like MCI. In contrast, another study [62] assessing attitudes toward VR training based on individual factors like gender, observed differing results from ours. For instance, while that study found a strong positive attitude in medical students toward VR-based teaching and assessment, female students exhibited comparatively lower positivity, indication potentially indicating gender differences that should be considered when implemented VR in the curriculum. Although the absence of significant impact at the item level in our study further underlines the generalisability of the positive influence at the group level, regardless of gender, occupation, experience and age, it remains crucial to address any potential disparities that may arise.

Contrary to the predominant focus on nursing staff in triage and management functions during MCI in reviewed studies [31, 36, 37, 39, 59, 63, 64], our results demonstrate that MFRs from various emergency health fields can acquire, train and manage MCI with positive impacts on their perception of self-efficacy. This broader applicability is crucial, as it indicates that any healthcare professionals activated in these situations can benefit from this type of training, enhancing overall response capability [19].

Hence, within the context of equipping healthcare professionals, particularly MFRs, to address the complexities presented in MCI, this study’s findings affirm the effectiveness of simulation-based training in enhancing self-efficacy, regardless of individual demographic variables. These findings emphasize the significance of recognizing self-efficacy as a pivotal metric, elucidating the training’s effectiveness in readying healthcare professionals for the intricate challenges associated with disaster scenarios.

In medical education, the focus often lies on teaching students’ essential knowledge and skills. However, more than 30 years of research on self-efficacy underscores that possessing knowledge and skills alone does not guarantee the motivation to apply them when needed [53]. From an educational perspective, assessing and positively influencing these self-efficacy trajectories is imperative, as individuals who persist through challenges actively strive for their self-efficacy trajectories throughout their clinical practice.

However, within the occupational context, an increase in the use of communications was noted between doctors and nurses. This observation is rationalized by their role in the SUMMA 112 service, where they assume responsibility for communication on ordinary devices during operational tasks. Regarding administering prescribed treatment at advanced medical posts, no differences in self-efficacy/perception were observed between doctors and nurses, as this skill aligns with their daily routine duties. Conversely, distinctions emerged among technicians, who exhibited heightened preparedness post-simulation. Our findings indicate that brief simulations contribute positively to self-efficacy, particularly for tasks less familiar in the routine scope of daily work. This underscores the potential of simulation-based training in enhancing self-efficacy, especially for healthcare professionals facing novel or less routine challenges.

## Conclusions

The study demonstrates the effectiveness of a comprehensive MCI training program in significantly enhancing participants’ overall self-efficacy and management skills. Through theoretical instruction, practical workshops, and simulation-based exercises, participants improved their ability to handle patient treatment, coordination, and communication during simulated MCI. These findings align with prior research on simulation-based training’s positive impact on self-efficacy beliefs. The study suggests that such training methodologies can boost healthcare professionals’ self-confidence, regardless of demographic variables. Unlike previous studies focusing predominantly on nursing staff, our findings show that MFRs from various emergency health fields can effectively manage MCI, enhancing their self-efficacy. In summary, the study highlights the value of simulation-based training in preparing healthcare professionals for disaster scenarios and emphasizes its potential to enhance self-efficacy, especially for less routine tasks.

### Limitations and future lines of research

With the implementation of this scale, we will be able to compare the levels of self-efficacy acquired using the different types of training:

- To assess training using new technologies such as virtual, mixed and augmented reality (under development), for which work is underway, to discover whether it is useful for improving this type of MCI training and participants’ self-efficacy.
- Evaluate the impact and effectiveness of training proposals specialized in self-efficacy to assess aspects related to developing competencies.
- To identify factors related to self-efficacy in personnel involved in MCI.
- Examine the relationship between self-efficacy and MCI performance.

### Supplementary Information

Supplementary Material 1: Annex I. Kirckpatrick levels of effective simulation training (Niemann, L. & Thielsch, M. (2020). Evaluation of Basic Trainings for Rescue Forces) [65]. Annex II. Timetable for the MCI Course Summa Procedure.

## Data Availability

Raw data from this study can be made available at reasonable request from the authors. https://docs.google.com/spreadsheets/d/1mKfv-kVSs3lj9qrvojbfybU7Os3xddo6/edit?usp=drive_link&ouid=106466966080904205286&rtpof=true&sd=true
