# Supplementary material for: Use of Self-Efficacy Scale in Mass Casualty Incidents During Drill Exercises
Source: BMC Health Serv Res. 2024 Jun 18;24:745. doi: 10.1186/s12913-024-11175-w (PMC11184813; doi:10.1186/s12913-024-11175-w)
Supplement: Supplementary file 1 — Supplementary Material 1: Annex I. Kirckpatrick levels of effective simulation training (Niemann, L. & Thielsch, M. (2020). Evaluation of Basic Trainings for Rescue Forces) [65]. Annex II. Timetable for the MCI Course Summa Procedure. [file 12913_2024_11175_MOESM1_ESM.docx]

**ANNEX I: Kirckpatrick levels of effective simulation training (Niemann, L. & Thielsch, M. (2020). Evaluation of Basic Trainings for Rescue Forces)**


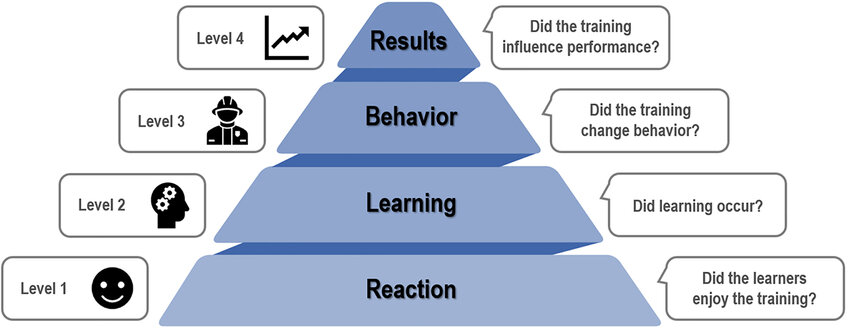


**ANNEX II: TIMETABLE FOR THE MCI COURSE SUMMA PROCEDURE**

**DAY 1**

| **SCHEDULE** | **WORKSHOP** | **CONTENTS** |
| --- | --- | --- |
| **9:30-9:45** | **Presentation of the course** | **Reception of students.**  **Welcome.** |
| **9:45-10:45** | **MCI SUMMA procedure** | **Technical instruction**  **Annex Barajas**  **Other institutions** |
| **10:45-11:00** | **Questions** |  |
| **11:00-12:00** | **Roles & Vests** | **Location and legends**  **Role functions**  **Supporting documentation** |
| **12:00-12:30** | **BREAK** |  |
| **12:30-13:30** | **Triage and backpack** | **Definition of out-of-hospital triage.**  **Triage in the different phases of MCI: DUAL, START/ Jump Start.**  **Triage backpack: location and content.**  **Triage card.**  **Assistance backpack: location and contents.**  **Support documentation.** |
| **13:30-14:15** | **Communications** | **Communications procedure**  **Channel management and operation**  **Broadcast priority and radio language** |
| **14:15-14:30** | **Course closure** | **Questions and Answers**  **Post-test**  **DPost-testion and links**  **Closing and farewell** |

**DAY 2**

| **SCHEDULE** | **WORKSHOP** | **CONTENTS** |
| --- | --- | --- |
| **9:00-9:30** | **Introduction to the SELF-EFFICACY STUDY** | **Consent and pretest instructions** |
| **9:30-10:30** | **Table-top simulation** | **Case studies will be carried out in small groups using blackboards and magnets.** |
| **10:30-12:00** | **Students will be divided into two stations** | **Triage card workshop**  **Communications and roles** |
| **12:00-12:30** | **BREAK** |  |
| **12:30-13:15** | **Brief Simulation** | **MCI activation (students only)** |
| **13:15-13:30** | **Briefing** |  |
| **13:30-14:15** | **Brief Simulation** | **MCI activation (students only)** |
| **14:15-14:45** | **SELF-EFFICACY STUDY** | **Postest** |
| **14:45-15:00** | **QR code signature** | **END OF COURSE** |
